# Intrinsic Brain Activity of Inferior Temporal Region Increased in Prodromal Alzheimer's Disease With Hearing Loss

**DOI:** 10.3389/fnagi.2021.772136

**Published:** 2022-01-28

**Authors:** Luwei Hong, Qingze Zeng, Kaicheng Li, Xiao Luo, Xiaopei Xu, Xiaocao Liu, Zheyu Li, Yanv Fu, Yanbo Wang, Tianyi Zhang, Yanxing Chen, Zhirong Liu, Peiyu Huang, Minming Zhang

**Affiliations:** ^1^Department of Radiology, The 2nd Affiliated Hospital of Zhejiang University School of Medicine, Hangzhou, China; ^2^Department of Neurology, The 2nd Affiliated Hospital of Zhejiang University School of Medicine, Hangzhou, China; ^3^Department of Neurology, Tongde Hospital of Zhejiang Province, Hangzhou, China

**Keywords:** hearing loss, Alzheimer's disease, mild cognitive impairment, resting-state functional MRI, fractional amplitude of low-frequency fluctuation

## Abstract

**Background and Objective:**

Hearing loss (HL) is one of the modifiable risk factors for Alzheimer's disease (AD). However, the underlying mechanism behind HL in AD remains elusive. A possible mechanism is cognitive load hypothesis, which postulates that over-processing of degraded auditory signals in the auditory cortex leads to deficits in other cognitive functions. Given mild cognitive impairment (MCI) is a prodromal stage of AD, untangling the association between HL and MCI might provide insights for potential mechanism behind HL.

**Methods:**

We included 85 cognitively normal (CN) subjects with no hearing loss (NHL), 24 CN with HL, 103 mild cognitive impairment (MCI) patients with NHL, and 23 MCI with HL from the ADNI database. All subjects underwent resting-state functional MRI and neuropsychological scale assessments. Fractional amplitude of low-frequency fluctuation (fALFF) was used to reflect spontaneous brain activity. The mixed-effects analysis was applied to explore the interactive effects between HL and cognitive status (GRF corrected, voxel *p*-value <0.005, cluster *p*-value < 0.05, two-tailed). Then, the FDG data was included to further reflect the regional neuronal abnormalities. Finally, Pearson correlation analysis was performed between imaging metrics and cognitive scores to explore the clinical significance (Bonferroni corrected, *p* < 0.05).

**Results:**

The interactive effects primarily located in the left superior temporal gyrus (STG) and bilateral inferior temporal gyrus (ITG). *Post-hoc* analysis showed that NC with HL had lower fALFF in bilateral ITG compared to NC with NHL. NC with HL had higher fALFF in the left STG and decreased fALFF in bilateral ITG compared to MCI with HL. In addition, NC with HL had lower fALFF in the right ITG compared to MCI with NHL. Correlation analysis revealed that fALFF was associated with MMSE and ADNI-VS, while SUVR was associated with MMSE, MoCA, ADNI-EF and ADNI-Lan.

**Conclusion:**

HL showed different effects on NC and MCI stages. NC had increased spontaneous brain activity in auditory cortex while decreased activity in the ITG. Such pattern altered with disease stage changing and manifested as decreased activity in auditory cortex along with increased activity in ITG in MCI. This suggested that the cognitive load hypothesis may be the underlying mechanism behind HL.

## Introduction

Alzheimer's disease (AD) is the most primary cause of dementia, which is clinically characterized by progressive cognitive decline (Scheltens et al., 2021). Given the enormous burden of AD on family nursing and public health, early identification of AD and timely management are particularly imperative (Livingston et al., 2020). On the one hand, as a transitional stage between normal aging and AD, mild cognitive impairment (MCI) is an appropriate period for early diagnosis and prevention of AD. On the other hand, around 9% of dementia cases suffer from hearing loss (HL), which has the highest population attributable fraction for dementia (Livingston et al., 2020). Consequently, understanding the association between HL and MCI may potentially provide a hint on the early management of AD. Several studies have indicated the relationship between HL and cognitive decline, and the role of HL as an increased risk for dementia (Deal et al., 2017; Thomson et al., 2017; Wei et al., 2017; Zheng et al., 2017). For example, David G. Loughrey found that age-related HL was related to increased risk of dementia (Loughrey et al., 2018). Similarly, a longitudinal study of 836 subjects found that elders with HL had an higher risk of developing dementia and a faster decline of cognition (Gurgel et al., 2014). Cognitive decline was often accompanied by the alternation in brain structure and function. Further evidence can be found in several previous morphological neuroimaging studies. For example, HL subjects suffer from microstructural dysfunction of auditory fiber tracts (Chang et al., 2004; Golub, 2017) and more rapid development of brain atrophy, especially in the temporal lobe (Eckert et al., 2012; Lin et al., 2014; Di Stadio et al., 2021). However, further research should be done to reflect the regional neuronal abnormalities.

Identification of regional neuronal abnormalities is crucial to understand the underlying mechanism behind neurodegenerative diseases. Resting-state functional magnetic resonance imaging (rsfMRI) is sensitive to imperceptible brain functional changes (Hohenfeld et al., 2018). Moreover, fractional amplitude of low-frequency fluctuation (fALFF) can effectively reflect spontaneous brain activities (Zou et al., 2008) thus has been widely applied in AD related research (Yang et al., 2020; Marin-Marin et al., 2021). For example, Liu et al. observed the fALFF in AD spectrum and found that the progression of AD could be portrayed by abnormal spontaneous regional neural activity (Yang et al., 2020). In addition, 18F-fluorodeoxyglucose positron emission tomography (FDG-PET) is a reliable method to reflect regional neuron glycometabolism (Kato et al., 2016; Caminiti et al., 2018). Blazhenets et al. demonstrated that FDG-PET had a reliable predictive value for the progression of MCI patients (Blazhenets et al., 2020). Hence, the combination of fALFF and FDG-PET could better illustrate the local brain activity in prodromal AD with HL.

In this study, we aim to explore the effect of HL on spontaneous brain activity in early stage of AD by combining the fALFF with FDG-PET. According to previous studies (Martini et al., 2014; Fulton et al., 2015; Wayne and Johnsrude, 2015; Griffiths et al., 2020), the long-term effect of HL on cognition was negative, while the early effect was unclear. We thus hypothesize that (1) the spontaneous brain activity of cognitively normal (CN) and MCI populations may respond differently to HL; (2) such functional changes may correlate with cognition.

## Materials and Methods

### Alzheimer's Disease Neuroimaging Initiative

The data used in this article were from the Alzheimer's Disease Neuroimaging Initiative (ADNI) database (http://www.loni.ucla.edu/ADNI). The database was launched by the National Institute on Aging (NIA), the Food and Drug Administration (FDA), and the National Institute of Biomedical Imaging and Bioengineering (NIBIB), aiming to explore whether serial magnetic resonance imaging (MRI), positron emission tomography (PET), biological markers, and other neuropsychological assessment can be used for early detecting and tracking AD. Ascertain of specific and sensitive markers for early AD will assist clinicians and researchers in understanding AD better and developing novel treatments.

### Participants

**Figure 1:**
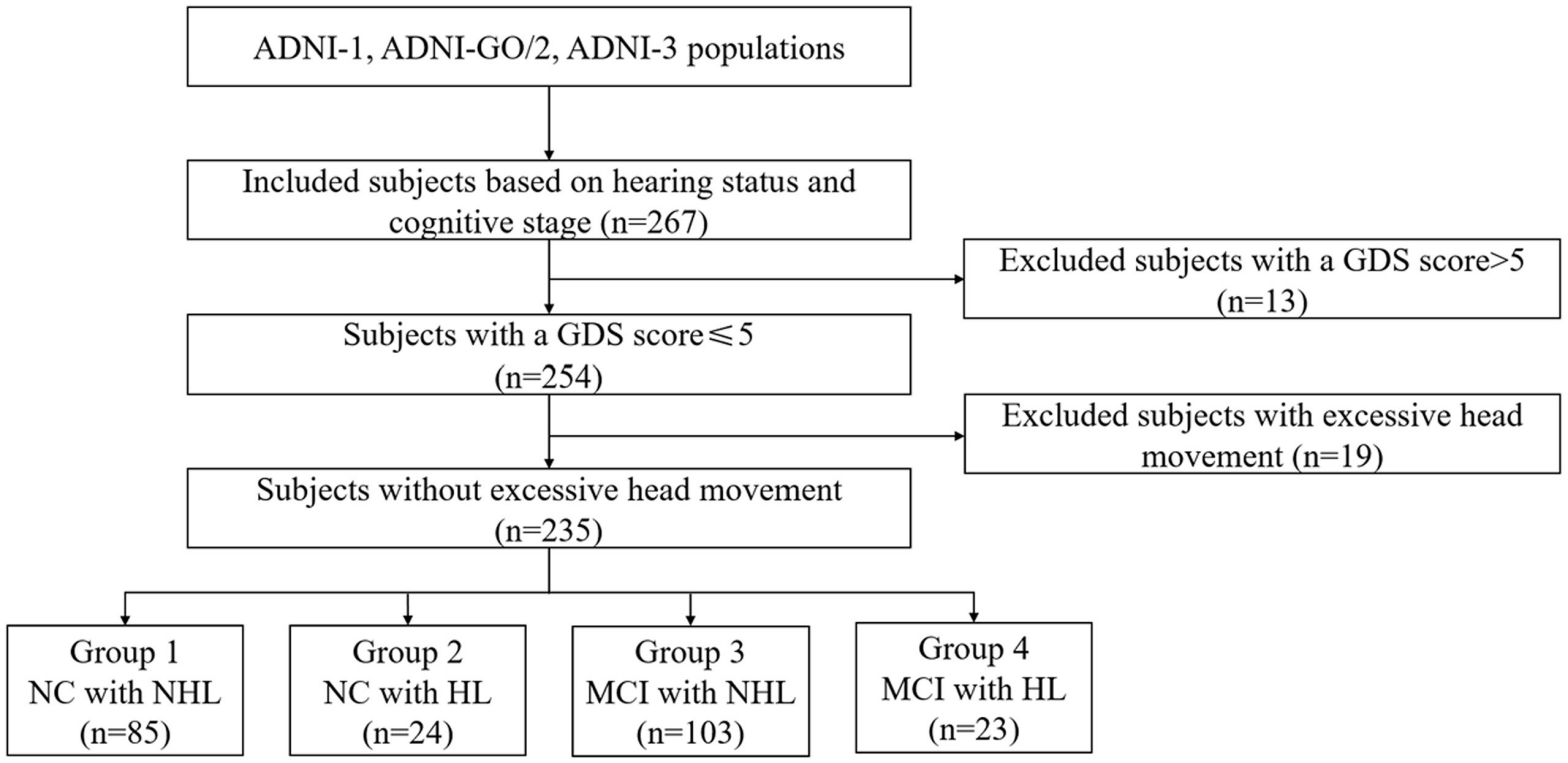
Schematic flow diagram of the sample size. NC, normal cognition; MCI, mild cognitive impairment; HL, hearing loss; NHL, no hearing loss; GDS, Geriatric Depression Scale.

Each participant signed the informed consent. All subjects underwent both T1-weighted 3D images, rsfMRI images, and neuropsychological scales. Notably, only CN and MCI were included in the current study since we aimed to explore the effect of HL on prodromal AD. Finally, we included 109 CN subjects and 126 MCI subjects (Figure 1; Supplementary 1).

The inclusion criteria for CN were: (1) a Clinical Dementia Rating (CDR) score of 0; (2) a Mini-Mental State Examination (MMSE) score between 24 and 30; and (3) without any report about cognition impairment. The criteria for MCI were: (1) a CDR score of 0.5; (2) an MMSE score between 24 and 30; (3) objective cognitive decline defined as scoring lower than the education-adjusted cut-off score on the delayed recall test of the Wechsler Memory Scale Logical Memory (WMS-LM); (4) preserved daily living ability; and (5) subjects insufficient to be diagnosed with dementia (Bondi et al., 2014).

Exclusion criteria were: (1) a Geriatric Depression Scale (GDS) score >5; (2) obvious traumatic encephalopathy; (3) serious mental or neurological illness; (4) abuse of alcohol or drugs; (5) medication history affecting brain function; and (6) left-handedness.

### Neuropsychological Scales Results

Neuropsychological scales included general cognitive state: MMSE, Montreal Cognitive Assessment (MoCA), Trail Making Test A (TMT-A), Trail Making Test B (TMT-B), memory function (ANDI-Mem), executive function (ADNI-EF), language (ADNI-Lan), and visuospatial function (ADNI-VS). Detailed information can be found in Supplementary 2.

### Group Classification

Similar to previous studies (Liu and Lee, 2019; Osler et al., 2019), we defined HL based on the medical report. Specifically, subjects with hearing loss or hearing impairment, referred in medical history, were defined as HL. The rests with no hearing loss were defined as NHL.

Then, we integrated the cognitive state and hearing status to classify all subjects into four groups: (1) Group 1, CN+NHL (*n* = 85); (2) Group 2, CN+HL (*n* = 24); (3) Group 3, MCI+NHL (*n* = 103); (4) Group 4, MCI+HL (*n* = 23). Notably, the FDG-PET data was only available in some of the patients since the examination is invasive. Thus, FDG-PET data included in the current study consists of 32 out of 85 in group 1, 12 out of 24 in group 2, 88 out of 103 in group 3 and 22 out of 23 in group 4.

### Image Acquisition

#### MRI

The structural images were obtained based on 3D Magnetization Prepared Rapid Acquisition Gradient Echo (MPRAGE) T1 weighted sequence, with followed parameters: repetition time (TR) = 2,300 ms; echo time (TE) = 2.98 ms; inversion time (TI) = 900 ms; slice thickness = 1.2 mm. The rsfMRI scans were obtained using an echo-planar imaging sequence with the following parameters: time points = 140; TR = 3,000 ms; TE = 30 ms; the number of slices = 48; slice thickness = 3.4 mm; spatial resolution = 3.31 × 3.31 × 3.31 mm^3^; matrix = 64 × 64. According to the ADNI database's human scan protocol, all participants should keep their eyes open and focus on a point in the mirror during the entire fMRI scan.

#### FDG-PET

FDG-PET imaging protocols were as follows, radiotracer dose: 4.5–5.5 mCi; scan start time post-injection: 30 min; scans and scan duration: 30 min, 6 × 5-min frames; randoms correction: Singles (not real-time subtraction); primary reconstruction method: Iterative (fully 3D Iter; not 3D FORE IR): 4 iterations; 20 subsets; Grid: 128 × 128; FOV: 256 mm (results in voxel size of 2.0 mm); slice thickness: 3.27 mm; smoothing filter: NONE or 0.0 (for all filter options: loop filter, post-filter and z-axis filter). Detailed FDG PET imaging protocols can be found at http://adni.loni.usc.edu/methods/documents/.

### MRI Data Pre-processing

The preprocessing of rsfMRI data was performed using the Data Processing and Analysis for (Resting-State) Brain Imaging (DPABI; http://rfmri.org/dpabi) (Yan et al., 2016) based on the Statistical Parametric Mapping 12 (SPM12; www.fil.ion.ucl.ac.uk/spm) on the MATLAB platform. Firstly, the first 10 volumes of functional images were removed to reduce the impact of the initial magnetic field inhomogeneity. The remaining 130 images were realigned for correction of timing differences between each slice and head motion (Friston 24 parameter). Then, structural images were segmented into gray matter (GM), white matter (WM), and cerebrospinal fluid (CSF) (Ashburner and Friston, 2005). Subsequently, based on rigid-body transformation, individual structural images were co-registered to the mean functional image and spatially transformed to the Montreal Neurological Institute (MNI) space, then resampled into 3 × 3 × 3 mm^3^ cubic voxels. In order to remove the nuisance signals, the Friston 24-parameter model (Friston et al., 1996) was applied to regress the effects of head motion (Yan et al., 2013). Then, to reduce cardiac and respiratory effects, other covariates, such as WM and CSF signals, were regressed out. The functional images were spatially smoothed with a Gaussian kernel of 6 × 6 × 6 mm^3^ full widths at half maximum to decrease spatial noise. Finally, data were scrubbed to further reduce motion-related artifacts by using a frame-wise displacement (FD) threshold of 0.5, with which “bad” time points were deleted.

In the preprocessing procedure, 19 subjects were excluded due to excessive head motion (> 3.0 mm maximum displacement in any of the x, y, or z directions or > 3.0° of any angular motion).

### The fALFF Calculation

The fALFF is the ratio of power spectrum of low-frequency to that of the entire frequency range. The fALFF was calculated by using the DPABI toolbox. In particular, the time series were first converted to a frequency domain with a fast Fourier transform, and the power spectrum was obtained. The square root of the power spectrum was computed at each frequency of the power spectrum, and the averaged square root was obtained across 0.01–0.08 Hz at each voxel. Finally, the fALFF was calculated as the ratio of the low-frequency power spectrum to the power spectrum of the entire frequency range (Zou et al., 2008; Yan et al., 2016).

### PET Data Processing

FDG PET preprocessing was performed using the PET-PVE12 (an SPM toolbox for partial volume effects correction in brain PET) (Gonzalez-Escamilla et al., 2017). Based on an adaptive maximum a posterior approach with partial volume estimation, the structural MRI image was firstly segmented into GM, WM, and CSF. Then, skull-stripped structural MRI image was created to be used for PET-MRI co-registration. After that, PET images were co-registered to the T1-weighted images from the same subjects using rigid-body transformations (rotations and translations). In order to correct the partial volume effects of PET images, a voxel-based method proposed by Müller-Gärtner et al. (MG) was applied (Müller-Gärtner et al., 1992). Here, the isotropic point spread function was set to 8 mm based on the effective image resolution of the ADNI FDG-PET data. Then, the voxel-wise PET standard uptake value ratio (SUVR) map was calculated using the whole cerebellar signal in the raw PET images as the reference. For the sake of voxel-based analyses, the corrected images were deformed into MNI space and smoothed with an 8 mm FWHM Gaussian kernel.

### Statistical Analysis

The analysis of variance (ANOVA) and Chi-squared tests were used for continuous (age, education, and GDS) and categorical data (gender, APOE ε4 status), respectively. Subsequently, *post-hoc* two-sample *t*-test was applied to analyze the difference between each group (*p* < 0.05).

The DPABI toolbox was used to perform statistical analyses of MRI data. Specifically, we performed a 2 × 2 mixed effect analysis and explored the interactive effect between hearing (HL and NHL) and cognitive status (CN and MCI). Age, education, and gender were employed as covariates. Then we performed the Gaussian random field (GRF) correction (voxel *p*-value <0.005, cluster *p*-value <0.05, two-tailed) to correct for multiple comparisons. Finally, we performed a *post-hoc* analysis between each group to reveal the difference (*p* < 0.05). In addition, we repeated the above analysis after adding APOE status (APOE ε4 carrier = 1, APOE ε4 non-carrier = 0) as a covariate.

Clusters showing significant between-group differences were defined as regions of interest (ROIs) and mean fALFF values were extracted accordingly. Mean FDG SUVR value from these ROIs were also obtained. Then, we performed Pearson correlation analysis between cognitive scores and fALFF values (Bonferroni corrected, *p* < 0.05). The associations between cognitive scores and FDG SUVR were also assessed (Bonferroni corrected, *p* < 0.05).

## Results

### Demographic and Clinical Characteristics

Detailed demographics are shown in Table 1. NHL group showed higher proportion of females compared to HL group, both in CN and MCI group (*p* < 0.001). MCI group showed lower age and higher GDS score compared to CN group with the same hearing state (*p* < 0.001). There is no group difference in education among four groups. For neuropsychological data, MCI+NHL group showed lower cognitive scores in MMSE (*p* < 0.001), MoCA (*p* < 0.001), ADNI-Mem (*p* < 0.001), ADNI-EF (*p* < 0.001), and ADNI-Lan (*p* < 0.001) compared to CN+NHL. MCI+HL group had lower ADNI-Mem than CN+HL group (*p* < 0.001).

**Table 1:** Demographic information and neuropsychological test results of the four groups.

	**CN**		**MCI**		***p*-value**
	**NHL (*n* = 85)**	**HL (*n* = 24)**	**NHL (*n* = 103)**	**HL (*n* = 23)**	
Age (y)	76.91 ± 6.57	80.03 ± 6.39	73.22 ± 7.78	72.82 ± 7.59	<0.001^b^^d^^e^
Gender (M/F)	35/50 (41.18%)	19/5 (79.17%)	51/52 (49.51%)	19/4 (82.61%)	<0.001^a^^c^^d^^f^
Education (y)	16.65 ± 2.54	16.67 ± 2.17	15.96 ± 2.76	17.39 ± 2.54	0.067
APOE ε4 status (*n*)	21 (26.25%)	8 (33.33%)	36 (37.11%)	13 (59.10%)	0.036^c^
GDS	0.71 ± 0.91	0.71 ± 1.12	1.56 ± 1.38	2.00 ± 1.51	<0.001^b^^c^^d^^e^
CDR	0.00	0.00	0.50	0.50	-
MMSE	29.01 ± 1.34	28.58 ± 1.64	28.05 ± 1.78	27.74 ± 2.00	<0.001^b^^c^
MoCA	25.89 ± 2.66	25.08 ± 2.86	23.22 ± 3.54	22.91 ± 3.29	<0.001^b^^c^
ADNI-Mem	1.02 ± 0.55	0.82 ± 0.60	0.39 ± 0.67	0.30 ± 0.63	<0.001^b^^c^^d^^e^
ADNI-EF	0.97 ± 0.92	0.94 ± 0.90	0.41 ± 0.83	0.56 ± 0.98	<0.001^b^
ADNI-Lan	1.00 ± 0.69	0.78 ± 0.57	0.31 ± 0.81	0.45 ± 0.71	<0.001^b^^c^^d^
ADNI-VS	0.21 ± 0.63	0.09 ± 0.76	0.13 ± 0.68	−0.17 ± 0.98	0.094
TMT-A (s)	33.86 ± 12.06	31.96 ± 7.64	36.88 ± 14.20	35.82 ± 14.86	0.262
TMT-B (s)	88.18 ± 57.66	70.04 ± 25.13	107.53 ± 59.23	94.82 ± 57.02	0.014^d^

*Data are presented as mean ± SD or n (%)*.

*CN, cognitively normal; MCI, mild cognitive impairment; HL, hearing loss; NHL, no hearing loss; GDS, Geriatric Depression Scale; CDR, Clinical Dementia Rating; MMSE, Mini-Mental State Examination; MoCA, Montreal Cognitive Assessment; TMT-A, Trail Making Test A; TMT-B, Trail Making Test B*.

a *Group1 vs. Group 2*.

b *Group 1 vs. Group 3*.

c *Group 1 vs. Group 4*.

d *Group 2 vs. Group 3*.

e *Group 2 vs. Group 4*.

f *Group 3 vs. Group 4*.

### The fALFF Results

**Figure 2:**
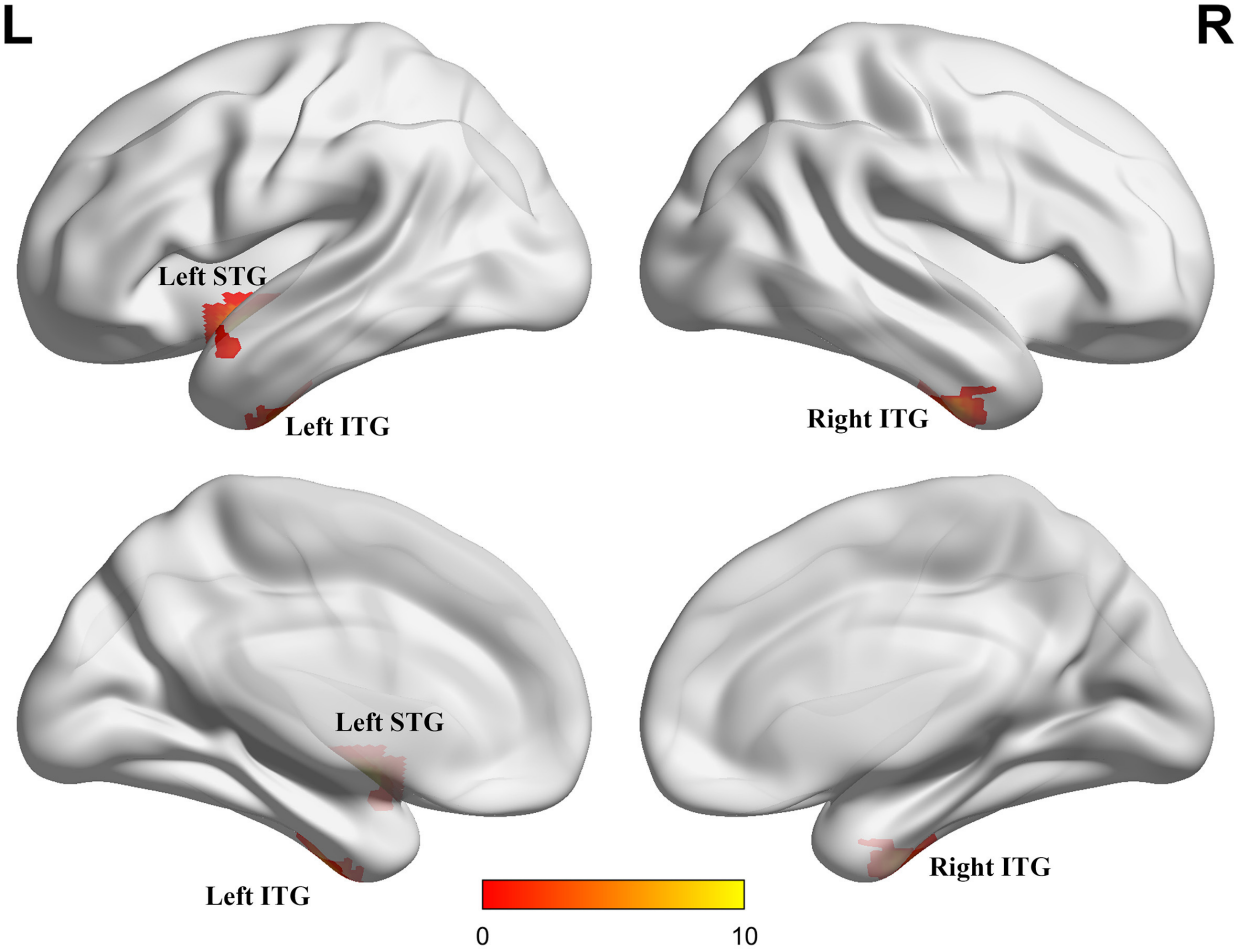
Three brain regions with significant differences in fALFF. These regions are the left superior temporal gyrus (left STG), the left inferior temporal gyrus (left ITG) and the right inferior temporal gyrus (right ITG), respectively. The results were obtained by analysis of covariance (ANCOVA) analysis adjusted with mean age, gender, education and mean frame-wise displacement [FD; *P* < 0.005, cluster level < 0.05, two-tailed, Gaussian random field (GRF) correction].

**Figure 3:**
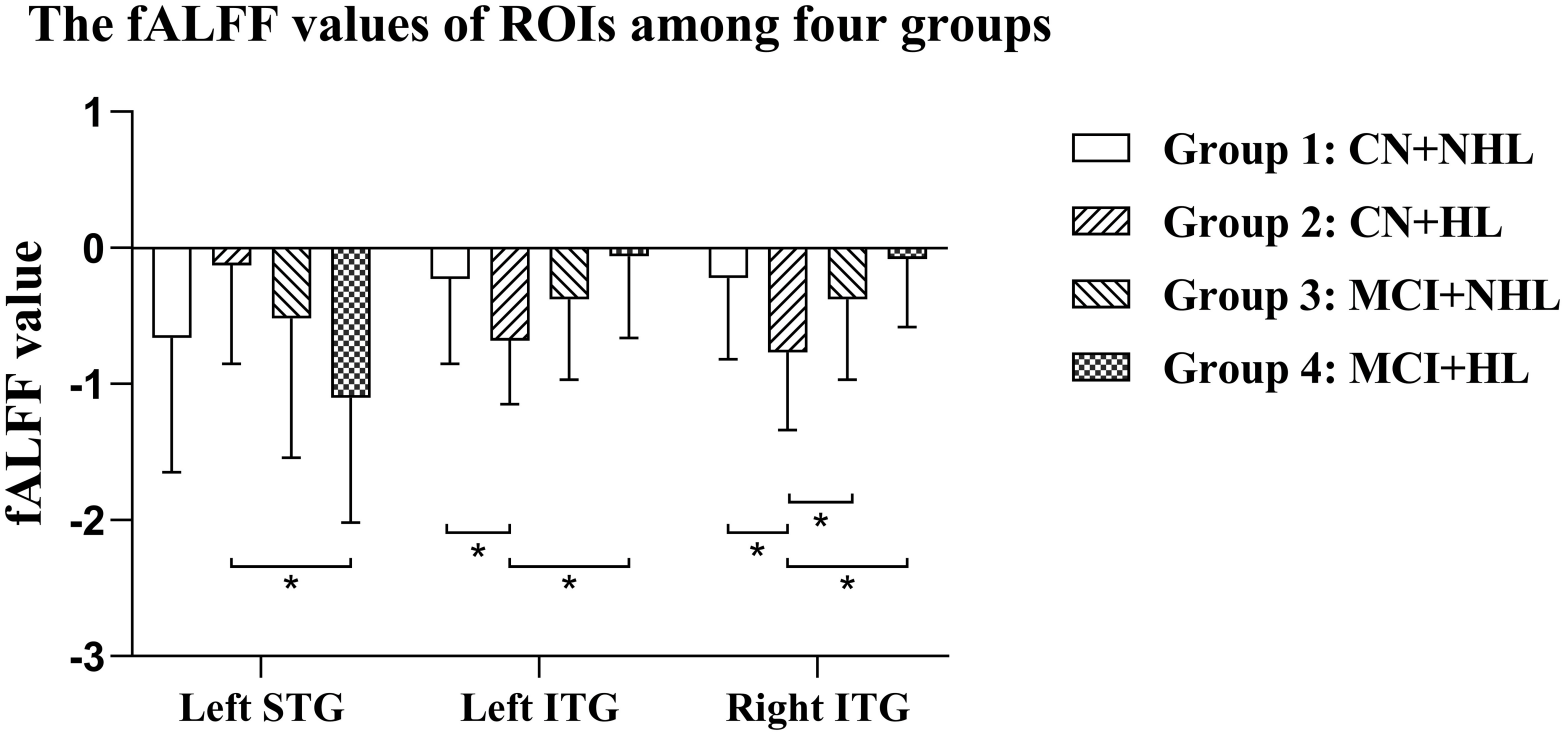
Specific differences in fALFF values in three brain regions among four groups. Left STG, the left superior temporal gyrus; Left ITG, the left inferior temporal gyrus; Right ITG, the right inferior temporal gyrus. *Means statistical significance level of *p* < 0.05/6, Bonferroni.

ANCOVA showed that the interactive effects primarily located in the left superior temporal gyrus (STG) and bilateral inferior temporal gyrus (ITG) (Figure 2; Table 2). Results remained constant after adding APOE status as a covariate (Supplementary Table 1). *Post-hoc* analysis showed that CN+HL had lower fALFF in bilateral ITG compared to CN+NHL (*p* < 0.05, Bonferroni corrected). CN+HL showed increased fALFF in the left STG and decreased fALFF in bilateral ITG compared to MCI+HL (*p* < 0.05, Bonferroni corrected). In addition, CN+HL showed decreased fALFF in the right ITG compared to MCI+NHL (*p* < 0.05, Bonferroni corrected) (Table 3; Figure 3).

**Table 2:** Details of three brain regions where HL and cognition interact.

**Brain region**	**Peak MNI coordinate**			**Peak intensity**	**Number of voxels**
	**X**	**Y**	**Z**		
Left STG	−42	0	−15	29.72	27
Left ITG	−45	−12	−33	17.14	15
Right ITR	57	−3	−36	15.73	14

*STG, superior temporal gyrus; ITG, inferior temporal gyrus; MNI, Montreal Neurological Institute*.

*The statistical threshold was set at p <0.005 with a cluster-level p <0.05 (two tailed, GRF corrected)*.

**Table 3:** The fALFF values of brain regions where HL and cognition interact.

**Brain region**	**Group 1**	**Group 2**	**Group 3**	**Group 4**	***p*-value**
Left STG	−0.66 ± 0.99	−0.13 ± 0.72	−0.52 ± 1.02	−1.10 ± 0.92	0.007^c^
Left ITG	−0.23 ± 0.62	−0.68 ± 0.47	−0.38 ± 0.59	−0.06 ± 0.60	0.002^a^^c^
Right ITG	−0.22 ± 0.60	−0.77 ± 0.57	−0.38 ± 0.59	−0.08 ± 0.50	<0.001^a^^b^^c^

*Data are presented as mean ± SD*.

*Group 1, CN+NHL; Group 2, CN+HL; Group 3, MCI+NHL; Group 4, MCI+HL*.

*STG, superior temporal gyrus; ITG, inferior temporal gyrus*.

a *Group 1 vs. Group 2*.

b *Group 2 vs. Group 3*.

c *Group 2 vs. Group 4*.

### Correlation Analysis Results

Detailed correlation analysis results are shown in Supplementary Table 2.

To explore the clinical significance of alterations in imaging related metrics, we performed the correlation analysis between cognitive performance and neuroimaging index. The fALFF in the left STG was negatively correlated with MMSE (*r* = −0.135, *p* < 0.05). The fALFF in the right ITG was negatively correlated with ADNI -VS (*r* = −0.143, *p* < 0.05).

To explore the possible mechanism behind these associations, we performed a correlation analysis between FDG-PET and the neuropsychological scale. Higher SUVR of the left STG was associated with higher MoCA score (*r* = 0.172, *p* < 0.05) and ADNI-Lan (*r* = 0.172, *p* < 0.05). Lower SUVR of the left ITG was associated with Lower MMSE score (*r* = 0.164, *p* < 0.05) and ADNI-EF (*r* = 0.203, *p* < 0.05). Moreover, the SUVR of the right ITG was significantly correlated with MMSE (*r* = 0.189, *p* < 0.05), ADNI-EF (*r* = 0.180, *p* < 0.05), TMT-A (*r* = −0.168, *p* < 0.05) and TMT-B (*r* = −0.189, *p* < 0.05).

## Discussion

Our study investigated the relations between HL and cognitive status reflected by spontaneous brain activity *in vivo*. We found a strong interactive effect between HL and cognitive status in the left STG and bilateral ITG. Further analysis showed that HL has different effects on CN and MCI. CN had increased spontaneous brain activity in auditory cortex and decreased activity in ITG. Such pattern altered with disease stage changing, demonstrated as decreased activity of the auditory cortex and increased activity in ITG in MCI. In general, HL and cognition do have an interactive effect on brain function, which may serve as the underlying mechanism of cognitive decline in the HL population. Our study may provide insights for the underlying mechanism behind HL in AD and suggest that early treatment of HL may postpone the transition of dementia.

The interactive effect (HL × cognitive status) we found mainly located in the regions involved in auditory (left STG) and visuospatial function (bilateral ITG). STG is the auditory cortex center (Tasaka et al., 2020) which receives auditory information from bilateral sources (mainly contralateral) (Brewer and Barton, 2016). Previous studies have shown that STG is mainly involved in extracting linguistic traits from speech input (Lin et al., 2014; Chern and Golub, 2019; Yi et al., 2019). In addition, previous studies found that brain atrophy, especially in STG, was associated with HL (Lin et al., 2014; Di Stadio et al., 2021). Similarly, a diffusion imaging study showed that there is a relationship between the left STG volume and auditory hallucination proneness (Spray et al., 2018). All these evidences support our findings to some extent. HL may be associated with degeneration of the auditory cortex. It is worth noting that above-mentioned results were only observed in the left STG but not the right side, which may indicate the spatial lateralization and related to the laterality of speech production (Kell et al., 2011; Hodgson and Hudson, 2018; Quass et al., 2018). This auditory lateralization was proposed by Christian Alexander Kell, who reported that speech production was a lateralized brain activity that could arise from a left dominance either in speech executive or sensory processes or both (Kell et al., 2011). Another study found that thickness of the left STG was correlated with cognitive performance, executive function, information processing speed, and attention (Achiron et al., 2013). Moreover, we also found a significant interactive effect (HL × cognitive status) on ITG, which is generally thought to be used to process visual information (Onitsuka et al., 2004) and multimodal sensory integration (Onitsuka et al., 2004). Some studies have also suggested that ITG may be involved in the integration of auditory information (Alain et al., 2018; Manno et al., 2021).

The cognitive load hypothesis may explain the functional changes that occur in these two brain regions. This hypothesis theorizes that HL may lead to the degradation of auditory signals, greater cognitive resources being required for auditory perceptual processing, and transfer from other cognitive tasks to effortful listening, ultimately resulting in cognitive reserve depletion (Fulton et al., 2015; Uchida et al., 2019). STG and ITG may be involved in auditory reception (Adank, 2012; Alain et al., 2018), processing (Du et al., 2016; Eckert et al., 2016), and integration (Onitsuka et al., 2004). In patients with HL, these regions may work together to keep auditory perception and cognition performance, and working patterns may change with the severity of the disease. Our results demonstrated a distinct effect pattern of HL on CN and MCI.

As for CN, CN+HL showed decreased fALFF in bilateral ITG, and an increasing trend of fALFF in STG compared to CN+NHL, indicating impaired function of information input. HL may lead to decline in speech input (Peelle et al., 2011; Eckert et al., 2012). CN subjects with HL may compensate the ability to process auditory information so as to maintain a relatively normal hearing experience (Pichora-Fuller et al., 1995; Tun et al., 2009). Nevertheless, compensation mechanism requires occupying global cognitive resources, which may lead to decreased activity in other brain regions (Tun et al., 2009; Martini et al., 2014). As a result, decreased activity in these occupied brain regions may lead to a decline in related cognitive performance, such as vision, attention, and working memory (Tun et al., 2009; Lin and Albert, 2014; Wayne and Johnsrude, 2015). The results also support the idea that the increased activity in local brain regions is actually the result of compensation. Our results showed that the fALFF value in the left STG was negatively correlated with MMSE, and the fALFF value in the right ITG was negatively correlated with visuospatial function. Since STG and ITG are spatially close to each other and both play a role in auditory processing and integration (Onitsuka et al., 2004), STG activity increased to compensate for the loss while ITG activity decreases when HL is present (Martini et al., 2014; Gagné et al., 2017). According to a study, the auditory processing regions, such as STG, are recruited by the visual system (ITG) when auditory input lost due to auditory deprivation (Manno et al., 2021). As mentioned above, the cognitive load theory may be the underlying mechanism between HL in MCI. Nevertheless, such pattern may change along AD stage according to our result.

As for MCI stage, MCI+HL showed increased fALFF in ITG and decreased fALFF in STG compared to CN+HL. This suggested decreased neuronal activity in STG of the MCI population compared to the CN population, while the neuronal activity in ITG is increased when HL is present. Such result could be explained by the effect of compensation. Generally speaking, long-term HL is always related to degeneration of the auditory cortex neuron (Chatani et al., 2016). Aforementioned compensatory effects can partly alleviate the effects of HL, but it might aggravate the burden on neurons accordingly, and the burden may start early and causing irreversible damage to neurons (Boyle et al., 2008). This explains the lower STG activity of MCI population compared to CN population. Despite this increased activity, widespread brain regions are activated during compensation to improve hearing experience (Pichora-Fuller et al., 1995; Griffiths et al., 2020). Studies have shown that compared to younger people, healthy older people requires broader brain activation to understand sentences of the same difficulty (Wingfield and Grossman, 2006; Vaden et al., 2015). This suggested that older people may have poorer auditory cortex function than younger people due to aging thus requires more cognitive resources for auditory information processing (Wingfield and Grossman, 2006).

As expected, we found that the fALFF value was adversely correlated with the FDG SUVR value in the left ITG. Although local neuronal activity is enhanced, some neurons in the same regions have been damaged at the same time due to overload. Studies have shown that in the case of long-term over-occupation, the function of related brain regions might be affected (Wingfield and Grossman, 2006). In addition, we also found the FDG SUVR value in STG was positively correlated with MoCA and language, and the FDG SUVR value in ITG was positively correlated with MMSE, executive function. As many studies have shown, HL is associated with cognitive decline, including but not limited to, general cognition, executive function and memory (Campbell and Sharma, 2013; Rönnberg et al., 2013; Gurgel et al., 2014).

There are several limitations to this study. Firstly, our sample size was relatively small. Due to the limited sample size, the current results should be treated with caution. Secondly, because the study was a cross-sectional design, the causal relationship between HL and cognition decline is unclear yet. But our results also provide novel insights into the mechanism between HL and cognition decline. It is necessary to repeat the investigation in a larger and longitudinal sample in the future. Thirdly, most participants with HL were male. This may be due to the gender distribution of HL. Some studies have shown that there is a gender difference in the incidence of hearing loss, such as higher incidence (Mitchell et al., 2011; Jafari et al., 2019) and earlier onset in males (Pearson et al., 1995). Nevertheless, gender has been adjusted as a covariate in current study. It is necessary to conduct research in the female population in future. Finally, the information of comorbidities (e.g., tinnitus and hyperacusis) and auditory amplification was unavailable. Thus, we were unable to examine whether comorbidities or auditory amplification affected the association between HL and neurodegeneration. Since this is only an exploratory study at present, future studies will consider these influencing factors and conduct longitudinal studies to investigate the impact of HL in brain activity along the AD continuum, from normal aging to more severe clinical stages.

## Conclusion

Our study demonstrated that the interaction between HL and cognition could be reflected by spontaneous brain activity (STG and ITG). As mentioned above, HL may have different effects on NC and MCI stages. Cognitive load theory may be the underlying mechanism behind this. This compensatory effect might also be responsible for cognitive decline in people with HL. This work may provide insights for the underlying mechanism behind HL and AD, suggesting that early treatment of HL may be meaningful to prevent AD.

## Data Availability Statement

The datasets presented in this study can be found in online repositories. The names of the repository/repositories and accession number(s) can be found below: http://adni.loni.usc.edu/.

## Ethics Statement

All procedures performed in studies involving human participants were under the ethical standards of the Institutional and National Research Committee and with the 1964 Helsinki declaration and its later amendments or comparable ethical standards. Written informed consent was from all participants and authorized representatives, and the study partners before any protocol-specific procedures were carried out in the ADNI study. More details from http://www.adni-info.org.

## Author Contributions

LH, QZ, and XLuo designed the study and wrote the first draft of the manuscript. LH and KL analyzed the MRI data and wrote the protocol. LH and QZ collected clinical and MRI data. XX, XLiu, ZLi, YF, YW, TZ, YC, ZLiu, PH, and MZ assisted with research design and interpretation of results. All authors contributed to the final manuscript, read, and approved the final manuscript.

## Funding

This study was funded by National Key Research and Development Program of China (Grant No. 2016YFC1306600), National Natural Science Foundation of China (Grant Nos. 81901707, 82001766, and 81701337), the Fundamental Research Funds for the Central Universities (No. 2017XZZX001-01), and Zhejiang Medicine and Health Science and Technology Program (2018KY418). The data collection and sharing for this project were funded by the ADNI (National Institutes of Health Grant U01 AG024904) and DOD ADNI (Department of Defense Award No. W81XWH-12-2-0012). ADNI was funded by the NIA, the NIBIB, and through generous contributions from the following: AbbVie, Alzheimer's Association; Alzheimer's Drug Discovery Foundation; Bio Clinica, Araclon Biotech; Inc.; Biogen; Bristol-Myers Squibb Company; Cere Spir, Inc.; Eisai Inc.; Elan Pharmaceuticals, Inc.; Eli Lilly and Company; F. Hoffmann-La Roche Ltd., Euro Immun; and its affiliated company Genentech, Inc.; Fujirebio; IXICO Ltd.; GE Healthcare; Johnson & Johnson Pharmaceutical Research and Development LLC.; Janssen Alzheimer Immunotherapy Research and Development, LLC.; Lumosity; Lundbeck; Merck & Co., Inc.; MesoScale Diagnostics, LLC.; Neurotrack Technologies; NeuroRx Research; Novartis Pharmaceuticals Corporation; Pfizer Inc.; Servier; Piramal Imaging; Transition Therapeutics; and Takeda Pharmaceutical Company. The Canadian Institutes of Health Research provides funds to support ADNI clinical sites. Private sector contributions are facilitated by the Foundation for the National Institutes of Health (www.fnih.org). The grantee organization is the Northern California Institute for Research and Education, and the study is coordinated by the AD Cooperative Study at the University of California, San Diego.

## Conflict of Interest

The authors declare that the research was conducted in the absence of any commercial or financial relationships that could be construed as a potential conflict of interest.

## Publisher's Note

All claims expressed in this article are solely those of the authors and do not necessarily represent those of their affiliated organizations, or those of the publisher, the editors and the reviewers. Any product that may be evaluated in this article, or claim that may be made by its manufacturer, is not guaranteed or endorsed by the publisher.
