# Supplementary material for: Intrinsic Brain Activity of Inferior Temporal Region Increased in Prodromal Alzheimer's Disease With Hearing Loss
Source: Front Aging Neurosci. 2022 Jan 28;13:772136. doi: 10.3389/fnagi.2021.772136 (PMC8831745; doi:10.3389/fnagi.2021.772136)
Supplement: Supplementary file 1 [file Data_Sheet_1.docx]

Supplementary Materials

# Supplementary Data

## Supplementary 1

The process of participant inclusion and exclusion.

According to study forms obtained from the ADNI website, we selected subjects from the ADNI 2, ADNI GO and ADNI 3 populations. Firstly, we screened 267 subjects from the ADNI database based on hearing status and cognitive stage. Secondly, given the effect of depression on cognition, we excluded 13 subjects with a GDS score greater than 5 (3 of Group 1, 1 of Group 2, 8 of Group 3, 1 of Group 4). After that, in the image preprocessing process, given the negative impact on image analysis caused by head movement, we ruled out 19 subjects with excessive head movement (10 of Group 1, 4 of Group 3, and 5 of Group 4).

We ended up with 235 subjects, 1) Group 1, NC with NHL (n=85); 2) Group 2, NC with HL (n=24); 3) Group 3, MCI with NHL (n=103); 4) Group 4, MCI with HL (n=23).

## Supplementary 2

The details of memory function (ANDI-Mem), executive function (ADNI-EF), language (ADNI-Lan) and visuospatial function (ADNI-VS).

ADNI-Mem was obtained by recording some items. These items included RVLT (Trial 1, Trial 2, Trial 3, Trial 4, Trial 5, Interference, Immediate recall, 30 minute delay and Recognition), ADAS-Cog (Trial 1, Trial2, Trial 3, Recall, Recognition present and Recognition absent), Logical Memory (Immediate and Delay) and MMSE (Ball recall, Flag recall and Tree recall).

The model for ADNI-EF included Category Fluency-animals, Category Fluency-vegetables, Trails A and B, Digit span backwards, WAIS-R Digit Symbol Substitution, and 5 Clock Drawing items (circle, symbol, numbers, hands, time).

The tests that make up ADNI-Lan included Neuropsychological Battery(Category Fluency-Animals, Category Fluency-Vegetables and Boston Naming), ADAS-Cognitive Behavior (Following Commands, Object Naming and Ideational Practice), MMSE (Naming an Object–Watch, Naming an Object–Pencil, Repeating a Sentence, Reading a Sentence, Writing a Sentence, Following a Series of Instructions) and MoCA (Letter F Fluency, Animal Naming-Lion, Camel, Rhino and Sentence Repetition).

ADNI-VS was consisted by Neuropsychological Battery (Clock copy–Circle, Clock copy–Symmetry, Clock copy–Numbers, Clock copy–Hands, Clock copy–Time), ADAS-Cognitive Behavior (Constructional praxis) and MMSE (Copy design).

# Supplementary Table

| **Supplementary Table 1**. The detail of three brain regions where hearing loss and cognition interact | | | | | |
| --- | --- | --- | --- | --- | --- |
| Brain region | Peak MNI coordinate | | | Peak intensity | Number of voxels |
|  | X | Y | Z |  |  |
| Left STG | -42 | 0 | -15 | 27.33 | 13 |
| Left ITG | -45 | -12 | -33 | 24.32 | 16 |
| Right ITR | 57 | -3 | -36 | 19.17 | 13 |
| Abbreviation: STG, superior temporal gyrus; ITG, inferior temporal gyrus; MNI, Montreal Neurological Institute  The statistical threshold was set at p﹤0.005 with a cluster-level p﹤0.05(two tailed, GRF corrected). | | | | | |

| **Supplementary Table 2.** Correlation between fALFF and FDG-PET SUV in three ROIs and neuropsychological scales | | | | | | | | | | | | | | | | | |
| --- | --- | --- | --- | --- | --- | --- | --- | --- | --- | --- | --- | --- | --- | --- | --- | --- | --- |
|  | fALFF-LSTG | |  | fALFF-LITG | |  | fALFF-RITG | |  | FDG-LSTG | |  | FDG-LITG | |  | FDG-RITG | |
|  | r | p |  | r | p |  | r | p |  | r | p |  | r | p |  | r | p |
| MMSE | -0.135 | 0.046^*^ |  | -0.063 | 0.356 |  | -0.017 | 0.805 |  | 0.109 | 0.187 |  | 0.164 | 0.047^*^ |  | 0.189 | 0.021^*^ |
| MoCA | 0.007 | 0.923 |  | -0.023 | 0.738 |  | -0.070 | 0.305 |  | 0.172 | 0.037^*^ |  | 0.054 | 0.515 |  | -0.060 | 0.466 |
| ADNI-Mem | 0.004 | 0.955 |  | 0.049 | 0.474 |  | 0.036 | 0.602 |  | 0.066 | 0.425 |  | 0.103 | 0.212 |  | 0.080 | 0.332 |
| ADNI-EF | -0.053 | 0.442 |  | -0.066 | 0.335 |  | -0.080 | 0.241 |  | 0.124 | 0.135 |  | 0.203 | 0.013^*^ |  | 0.180 | 0.029^*^ |
| ADNI-Lan | 0.002 | 0.975 |  | 0.073 | 0.283 |  | 0.081 | 0.236 |  | 0.172 | 0.036^*^ |  | 0.159 | 0.053 |  | 0.112 | 0.177 |
| ADNI-VS | 0.004 | 0.948 |  | -0.094 | 0.168 |  | -0.143 | 0.035^*^ |  | 0.017 | 0.841 |  | 0.017 | 0.835 |  | 0.050 | 0.543 |
| TMT-A (s) | -0.021 | 0.759 |  | 0.089 | 0.195 |  | 0.004 | 0.955 |  | 0.008 | 0.926 |  | -0.088 | 0.288 |  | -0.168 | 0.042^*^ |
| TMT-B (s) | -0.046 | 0.507 |  | 0.025 | 0.718 |  | 0.115 | 0.095 |  | -0.060 | 0.471 |  | -0.121 | 0.146 |  | -0.189 | 0.023^*^ |
| Abbreviation: fALFF-LSTG, fALFF value in the left superior temporal gyrus; fALFF-LITG, fALFF value in the left inferior temporal gyrus; fALFF-RITG, fALFF value in the right inferior temporal gyrus; FDG-LSTG, FDG-PET standard uptake value in the left superior temporal gyrus; FDG-LITG, FDG-PET standard uptake value in the left inferior temporal gyrus; FDG-RITG, FDG-PET standard uptake value in the right inferior temporal gyrus  ^*^ means significance level of p﹤0.05 | | | | | | | | | | | | | | | | | |
